# Statistical principle-based approach for recognizing and normalizing microRNAs described in scientific literature

**DOI:** 10.1093/database/baz030

**Published:** 2019-02-27

**Authors:** Hong-Jie Dai, Chen-Kai Wang, Nai-Wen Chang, Ming-Siang Huang, Jitendra Jonnagaddala, Feng-Duo Wang, Wen-Lian Hsu

**Affiliations:** 1 Department of Electrical Engineering, National Kaohsiung University of Science and Technology, Kaohsiung, Taiwan, ROC; 2 Big Data Laboratories, Chunghwa Telecom Co., Taoyuan, Taiwan, ROC; 3 Graduate Institute of Biomedical Electronics and Bioinformatics, National Taiwan University, Taipei, Taiwan; 4 Institute of Information Science, Academia Sinica, Taipei, Taiwan; 5 School of Public Health and Community Medicine, University of New South Wales, Sydney, Australia; 6 Department of Computer Science and Information Engineering, National Taitung University, Taitung, Taiwan

## Abstract

The detection of MicroRNA (miRNA) mentions in scientific literature facilitates researchers with the ability to find relevant and appropriate literature based on queries formulated using miRNA information. Considering most published biological studies elaborated on signal transduction pathways or genetic regulatory information in the form of figure captions, the extraction of miRNA from both the main content and figure captions of a manuscript is useful in aggregate analysis and comparative analysis of the studies published. In this study, we present a statistical principle-based miRNA recognition and normalization method to identify miRNAs and link them to the identifiers in the Rfam database. As one of the core components in the text mining pipeline of the database miRTarBase, the proposed method combined the advantages of previous works relying on pattern, dictionary and supervised learning and provided an integrated solution for the problem of miRNA identification. Furthermore, the knowledge learned from the training data was organized in a human-interpretable manner to understand the reason why the system considers a span of text as a miRNA mention, and the represented knowledge can be further complemented by domain experts. We studied the ambiguity level of miRNA nomenclature to connect the miRNA mentions to the Rfam database and evaluated the performance of our approach on two datasets: the BioCreative VI Bio-ID corpus and the miRNA interaction corpus by extending the later corpus with additional Rfam normalization information. Our study highlights and also proposes a better understanding of the challenges associated with miRNA identification and normalization in scientific literature and the research gap that needs to be further explored in prospective studies.

## Introduction

Research on MicroRNAs (miRNAs), endogenous small RNA molecules of about 22 nucleotides in length that can post transcriptionally regulate gene expression by base pairing to messenger RNAs, is one of the most widely discussed topics in science and medicine recently. The first miRNA was discovered over 30 years ago (1). Since then, miRNAs have been found to participate in many physiological and pathological processes. Numerous miRNAs and their potential targets have been identified by bioinformatics tools (2–4) and high-throughput sequencing (5–7). Therefore, the demand for monitoring scientific advancement and progress related to miRNA is increasing.

Validated miRNA targets are usually reported in literature. It can be estimated that the number of publications related to miRNA in PubMed will be over 19 600 in 2018. The rapidly increasing amount of miRNA-related literature provides researchers with abundant information but also makes it difficult to identify the literature of interest as well as keep up to date with the novel findings associated with miRNAs. Results and conclusions of studies on miRNA targets and their importance in many physiological processes can be retrieved using information extraction (IE) methods. These methods can be employed to extract miRNA-related information from the main body and the figure captions of the manuscript. The techniques established cannot only facilitate the construction of miRNA knowledge bases, but also enhance the index created by search tools and databases in obtaining more relevant literature using miRNA-specific keywords. With this in mind, we propose a statistical principle-based approach (SPBA) for miRNA recognition and normalization in full-text scientific articles. The performance of the developed method is assessed on two manually annotated corpora with miRNA terms and the corresponding Rfam database IDs.

## Related work

Web-based miRNA-related databases have been constructed for researchers to retrieve miRNAs and their target genes. For instance, miR2Disease (8) is a manually curated database providing a comprehensive resource for miRNA deregulation in various human diseases. It provides researchers with information such as miRNA–disease relationships and experimentally verified miRNA target genes, as well as references to the relevant biomedical literature. Similarly, the miRWalk database (9) provides predicted and validated miRNA binding site information related to miRNAs in humans, mice and rats. However, keeping these databases with up-to-date miRNA knowledge in a timely fashion is quite challenging due to the rapid growth of miRNA-related publications. Several databases such as miRSel (10) and miRCancer (11) have started applying IE approaches to automatically extract miRNA-related relations from literature.

Identifying miRNAs mentioned in text is one of the fundamental steps in the IE process of constructing miRNA knowledge databases. Rule-based (10–14) and supervised learning-based (15) approaches are two popular methods that have been used for this purpose. A few tools or web services were openly available and provided the functionality of identifying miRNA mentions from literature (12, 15–17). The miRNA nomenclature naming convention was formalized in the early 2000s (18). Thus, most of the previous studies manually developed rules based on regular expression patterns to recognize miRNA mentions. For example, Xie *et al*. (11) used prefix and suffix patterns to recognize miRNAs. The prefix patterns include terms frequently prefixed in miRNA terms, while the suffix patterns consist of terms for indicating hairpin precursor and hairpin loci information. Naeem *et al*. (10) compiled their patterns by examining synonyms and generic occurrences of miRNA names mentioned in various databases. These patterns also considered frequent spelling variants that appear in miRNA mentions, such as the omission of species identifiers (19). Murray *et al*. (20) developed sophisticated patterns to recognize miRNAs that consist of terms like ‘miR’, ‘mirn’, ‘mirna’ and ‘microRNA’. They claimed that the developed patterns can achieve 100% accuracy and recall against the miRBase (21). However, the corpus used in this study is not publicly available.

Bagewadi *et al*. (22) released the first ever openly available corpus annotated with miRNA terms and their relations with genes and diseases. They compiled their patterns for recognizing miRNAs based on the manually annotated corpus and achieved an average F-score of 0.9385 for the task of miRNA recognition. On the other hand, only a few studies employed the supervised learning-based approach for miRNA recognition. Lamurias *et al*. (15) adapted BANNER (http://banner.sourceforge.net/) to train a conditional random field model for recognizing miRNAs. Their approach obtained an F-score of 0.91 on the corpus released by Bagewadi *et al*.

Rule-based approaches require domain experts to manually develop rules, which may not be comprehensive to cover all miRNA naming variations, such as the insertion, deletion or substitution (IDS) of words appearing in the entities. By contrast, machine learning models can learn implicit patterns automatically, but the resulting model may not be interpretable by humans. Other disadvantages may include the reproducibility, transportability and portability of these models. The performance of the machine learning models depends heavily on the characteristics of the labeled training data such as size and representativeness. Another critical disadvantage of using rule-based or machine learning-based approaches is that additional work is required to normalize the recognized miRNAs to standard database IDs such as the ones used in Rfam. Some studies such as miRWalk (23) used a dictionary-based approach since the method can achieve both recognition and normalization at the same time. Balderas-Martínez *et al*. (13) further combined their dictionary-based approach with additional rules to improve the recall rates.

The proposed SPBA provides an integrated solution for miRNA recognition and normalization with the capability of overcoming the challenges and issues discussed. This approach has been successfully employed in several domains such as sentimental analysis and topic detection (24–26). Similar to the transformation-based learning (TBL) approach used in the Brill part-of-speech tagger (27), our SPBA method relied on patterns to recognize miRNA mentions and supervised learning approaches to induce patterns from an annotated corpora. Unlike TBL, which does not display the domain knowledge learned from the corpus into a human-interpretable manner, using SPBA we represented the knowledge by slots and organized them in a readable manner that can be further updated by domain experts.

The automatically induced patterns are composed of the slots learned from pre-labeled data and those manually created by domain experts. These patterns are considered as the dominated principles and their details are elucidated in the Methods section. Finally, SPBA employs a partial matching algorithm together with the principles to harness the advantages of both rule-based and supervised learning-based approaches while overcoming their limitations in recognizing miRNA mentions. Furthermore, similar to the dictionary-based approach, our SPBA-based normalization method can directly determine the most probable candidate ID based on the calculation of the scores of the matched slots following the matching process.

## Methods

Like supervised learning, SPBA involves two stages: training and prediction. The training phase of SPBA consists of two main steps. The first is knowledge construction, where SPBA uses a hierarchical principle slot combination scheme to express knowledge. For the task of recognizing miRNAs, a ‘principle’ refers to an organized semantic description of a miRNA. Each principle contains a collection of ‘slots’ and ‘relations’ specified among them. A slot, which may contain a set of words, phrases, semantic categories or other slots, serves as the basic component that holds a piece of information in a particular principle. One can specify relations like ordering and compatibility among slots in a principle.

Following knowledge construction is the principle generation step, which is similar to the learning process in TBL. In TBL, a set of rough tagging rules was used to tag the corpus, and the results were then iteratively improved by inducing new rules. In SPBA, we labeled the defined slots in the training set. These slots were then automatically assembled and summarized into principles by observing the arrangement of slots that can be used to recognize miRNA mentions. In the prediction stage, a given input text is also labeled with defined slots. Unlike TBL that applies an ordered list of transformation rules to generate the final part-of-speech tags, SPBA utilizes a matching algorithm allowing IDS within the learned principles to distinguish miRNA mentions. In the following subsections, we delineate the proposed SPBA for the miRNA identification task.

### Knowledge construction for miRNA recognition and normalization

**Figure 1:**
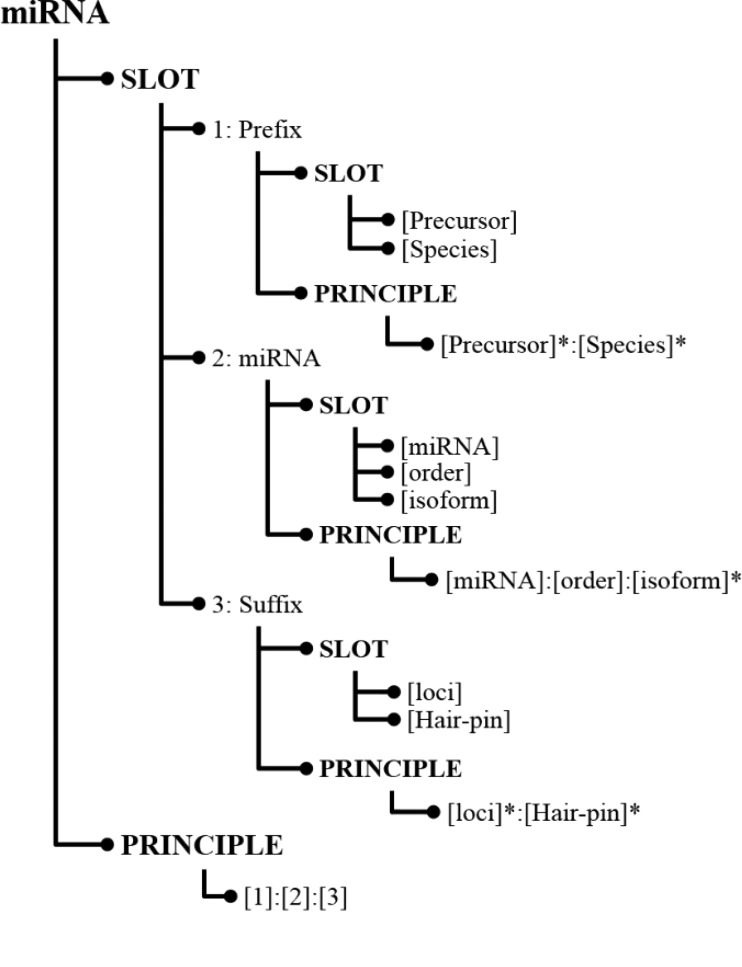
Knowledge represented for miRNA in InfoMap.

In SPBA, the principle slot scheme is represented in Information Map (InfoMap) (28). Figure 1 illustrates a simplified example of how the knowledge is constructed for representing a miRNA in InfoMap. The root node of the principle slot scheme in InfoMAP denotes the name of a domain or a subject, which is referred to as a ‘concept node’. The concept node ‘miRNA’ indicates that the node structure represents the knowledge for a miRNA name.

As shown in Figure 1, the first child node of a concept node is the ‘SLOT’ node in which we defined the fundamental slots for a miRNA mention. Albeit heterogeneous writing styles, some commonalities can be found among miRNA mentions, which were defined as slot nodes in InfoMap. For instance, both miRNAs ‘cel-miR-123-5p’ and ‘hsa-microRNA-24-3P’ consist of a species term (cel and hsa), the indicating word (miR and microRNA) and a hairpin that possess unique feature in representing a miRNA. Hence, we can define slots like ‘[Species]’ that encodes the species in which the miRNA appears and ‘[miRNA]’ representing the word indicating an occurrence of a miRNA name. Slot definitions in InfoMap can be generalized by organizing them in a hierarchical structure. For example, the last two slots [(loci) and (Hair-pin)] in Figure 1 were generalized by the slot, ‘[Suffix]’, which can be used to differentiate distinctive types between miRNAs.

For each slot, terms that could be used in literature were collected from the training set and listed under that slot. For example, the instances of the ‘[Species]’ slot contain terms such as ‘hsa’ and ‘cel’. The indicating words for [miRNA] include ‘mir’, ‘let’, ‘lsy’, ‘micro RNA’ … etc.

### Principle generation for miRNA recognition

As illustrated in Figure 1, the last node at each level of the hierarchical principle slot scheme is the ‘PRINCIPLE’ node, which stores relations among the defined slots. The last principle ‘[1]:[2]:[3]’ indicates that the principle is composed of the ‘Prefix’ slot followed by the ‘miRNA’ slot and then the ‘Suffix’ slot. To generate miRNA principles from training instances, firstly the given text must be labeled with the defined slots. Unlabeled words were considered as insertions, and the remainder of the labeled sequence was regarded as a candidate principle for representing miRNAs. Subsequently, several slot combinations were generated. To reduce the number of generated principles and make principles more generalizable to a different genre, we considered the principle generation task as a dominating set problem and developed an algorithm to summarize all candidate principles into more representative principles. In this way, we can capture the majority of the concept variations using a small number of principles.

**Table 1:** Illustration of a dominant principle and some dominated principles in the miRNA corpus generated by SPBA

**Dominant principle**					
[Precursor] [Species]**[miRNA] [Order]**[Conj][Order] [Suffix]					
**Principles dominated by dominant principles:** [Species][miRNA][Order][Order], [Precursor][miRNA][Order][Order], [miRNA][Order][Suffix]					
**Examples matched with the above principles**					
**Slot name**	**Precursor**	**Species**	**miRNA**	**Order**	**Suffix**
**Example**					
hsa-miR-181b	-	hsa	miR	181b	-
cel-miR-16-2		cel	miR	16–2	-
pre-miR-149	pre	-	miR	149	-
cel-miR-16a1	-	cel	miR	16a1	-
miR-485-5p	-	-	miR	485	5p

**Figure 2:**
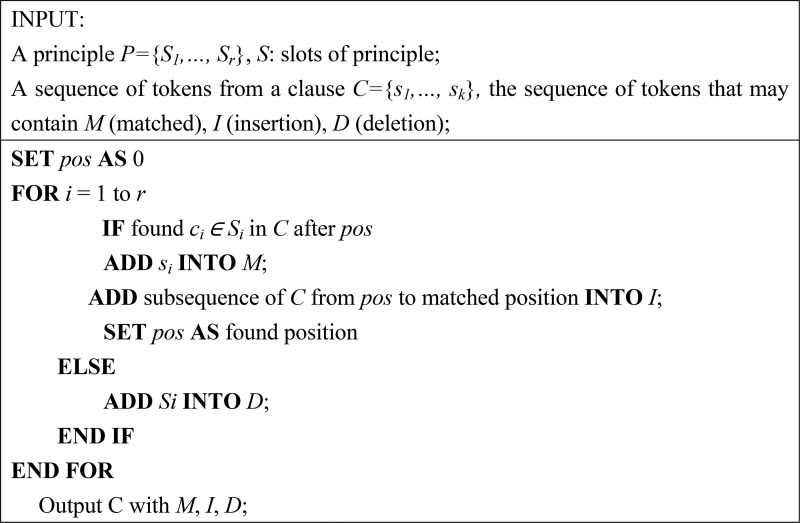
Principle matching algorithm.

However, it has been proven that finding a dominating set on a graph is nondeterministic polynomial time (NP)-hard (29). Thus, we implemented a greedy approximation algorithm as follows. First, we constructed a directed graph $G=\Big\{V,E\Big\}$, where *V* contains all candidate principles, and *E* represents the dominating relations among them. A dominating relation exists when a principle dominates another principle, so if a principle *p_i_* dominates another principle, *p_j_*, there is an edge starting from *p_i_* to *p_j_*. We used the criteria proposed in our previous work (30) to determine the dominating relations. Table 1 illustrates an example of the result of the principle generation step. It can be observed that the dominant principle and the dominated principles would cluster together when sharing certain key slots in common. Depending on the basis, a set of IDS values serves as the criteria to determine whether the principles in the same cluster are able to dominate each other or not. Once the conditions match the desired criteria, the cluster representative principle will dominate the other candidate principles.

### Principle matching for recognizing miRNAs

During the matching process for recognizing miRNAs mentioned in free text, the given sequence of words was first labeled with the compiled slots. We then employed an alignment-like algorithm depicted in Figure 2 to determine that the span of words that matched the principles defined in our InfoMap. Unlike normal handcrafted patterns, such as regular expressions in which rigid co-occurrence and ordering relations among slots must be defined, our SPBA compares the matched slots in a sequence of tokens (denoted as *C*) to the defined principles. The output of the algorithm includes the matched, insertion and deletion sets. For instance, the first dominated principle shown in Table 1 matches ‘miR-16-2’ by applying two insertions of ‘-’, while ‘pre-miR-149’ is matched with the second dominated principle with one deletion of the ‘Order’ slot and two insertions. Following the notations used in Figure 2, the matched, insertion and deletion sets are denoted as M, I and D, respectively.

Each set was associated with a different matching score, and the final matching score was calculated by using Equation 1, which utilizes all matched slots in *M*, and slot insertions/deletions in I/D as scoring criteria during the matching step. M, I and D were generated by the algorithm shown in Figure 2

(1) \begin{equation*} \mathrm{Score}\left(\mathrm{C}\right)=\sum_{S_i\in M}{\mathrm{S}\mathrm{core}}_m\left({\mathrm{S}}_i\right)-\sum_{S_j\in I,D}\mathrm{Score}\left({\mathrm{S}}_j\right) \end{equation*}

The score of the matched slot obtained from the probability of the slot belonging to a miRNA mention is calculated by Equation 2. In our implementation λ was set to 100.

(2) \begin{equation*} {\mathrm{S}\mathrm{core}}_{\mathrm{m}}\left({\mathrm{S}}_i\right)=\lambda \frac{freq_{miRNA}\left({\mathrm{S}}_i\right)}{freq_{miRNA}\left({\mathrm{S}}_i\right)+{freq}_{nonMiRNA}\left({\mathrm{S}}_i\right)} \end{equation*}

The score of insertion, defined as Equation 3, was calculated by the inversed entropy of the slot representing the uniqueness or generality of this slot being a miRNA mention. A deletion, defined in Equation 4, was computed from the log probability of the slot as a miRNA.

(3) \begin{align*} &{\mathrm{S}\mathrm{core}}_{\mathrm{i}}\left({\mathrm{S}}_i\right)\\&\quad=\nonumber\left\{\begin{array}{c}\frac{-1}{P_{miRNA}\left({\mathrm{S}}_i\right){\log}_2{P}_{miRNA}\left({\mathrm{S}}_i\right)+{P}_{nonMiRNA}\left({\mathrm{S}}_i\right){\log}_2{P}_{nonMiRNA}\left({\mathrm{S}}_i\right)}\\ {}\ \mathrm{if}\ P>0\kern1em \\ {}0\kern2em \mathrm{if}\ {P}_{nonMiRNA}=0\\ {}-\infty \kern2.5em \mathrm{if}\ {P}_{miRNA}=0\end{array}\right. \end{align*}

(4) \begin{equation*} {\mathrm{S}\mathrm{core}}_{\mathrm{d}}\left({\mathrm{S}}_i\right)=-{\log}_2{\mathrm{S}\mathrm{core}}_{\mathrm{m}}\left({S}_i\right) \end{equation*}

The threshold to accept a matched principle as a miRNA was determined by Equation (5).

(5) \begin{align*} &\mathrm{Threshold}\nonumber\\&\left(\left\{{\mathrm{S}}_1,\dots, {\mathrm{S}}_r\right\}\!,\left\{{\mathrm{S}}_{core_1},\dots, {\mathrm{S}}_{core_n}\right\}\!,\left\{{NonS}_1,\dots {NonS}_o\right\}\right)\nonumber\\ &\;={\sum}_{i=1}^n{\mathrm{S}\mathrm{core}}_{\mathrm{m}}\left({\mathrm{S}}_{core_i}\right)-\sum_{S_j\notin \left\{{S}_{core}\right\}}{\mathrm{S}\mathrm{core}}_{\mathrm{d}}\left({S}_j\right)\nonumber\\ &\quad+{\sum}_{k=1}^r\mathrm{P}\left({S}_k\right){\mathrm{S}\mathrm{core}}_{\mathrm{i}}\left({S}_k\right)+{\sum}_{l=1}^o\mathrm{P}\left({NonS}_l\right){\mathrm{S}\mathrm{core}}_{\mathrm{i}}\left({S}_l\right)\!, \end{align*}

where {*S_core_*} is the set of slots that appears in all dominated principles, {*S*} is the set of slots that appears in some dominated principles but not all and {*NonS*} is the set of words that may appear in a miRNA mention but not defined as slots. P(*S*) and P(*NonS*) are the probability of the slot belonging/not belonging to a miRNA mention, which were estimated by using the given corpus.

### Principle-based normalization

We extracted the following columns from the family file (downloaded from ftp://ftp.ebi.ac.uk/pub/databases/Rfam/CURRENT/database_files) to compile the lexicon for normalization:

- The first column: contains the family accession number (e.g. RF00994).
- The second column: contains the family id (e.g. mir-1255).
- The fourth column: contains the family description (e.g. miRNA mir-1255). For records containing the backslash character such as ‘mir-103/107 microRNA precursor’ for RF00129, we extracted terms like ‘mir-103’ and ‘mir-107’ semi-automatically by first using regular expressions and then manually verified the extracted results.
- The eleventh column: contains the previous family names (e.g. Y1, Y2, Y3 and Y5). Records with ‘\N’ were ignored.

We then used the generated principles to match all columns contained in miRNA names and built indexes for each slot. During the principle matching process, we scored the matched slots based on the matched principles over all entries in the compiled lexicon. Therefore, each slot will be associated with all possible corresponding grounding entries in our lexicon along with a matching score. For possible miRNA mentions recognized after the principle matching step, the indexes of the matched slots were used to effectively retrieve all possible grounding in the Rfam database. We then assign the mention with the normalization ID with the highest associated scores.

### Extended miRNA recognition and normalization corpus

We extended the miRNA interaction corpus (MIC) annotated by Bagewadi *et al*. (31) by manually assigning the Rfam ID to each annotated miRNA mention with the assistance of a dictionary-based exact matching method. The process cannot be fully implemented in an automatic fashion because of the variations of miRNA mentions [e.g. oncomir-1, mir-213a/b, let-7e and mirna (mir)-223] and mentions referring to multiple miRNAs like ‘mir-15/107’ and ‘mir-29a/b-1’. The original corpus contains 301 abstracts divided into the training and test sets. There are 1864 sentences in the training set and 780 sentences in the test set. Five bio-entity types including specific miRNA (e.g. has-miR-124b), non-specific miRNA (e.g. miRNAs), disease, gene and species were annotated. All of these annotations were annotated at the sentence level. In the training set, 327 sentences contain 529 specific miRNAs, while 376 specific miRNAs are included in the test set. After our annotation, the updated training dataset consisted of a total of 521 annotated miRNAs corresponding to 75 unique IDs from 1863 sentences within 201 articles. The test dataset comprises 780 annotated sentences from 100 articles with 375 annotations and 53 unique IDs. Some miRNA mentions were annotated with more than one ID, such as ‘RF00103, RF00446’ for the mention ‘miR-1/133a’.

**Table 2:** Statistics of the annotated miRNAs in the Bio-ID corpus

**Dataset**	**# of sentences/captions with miRNAs annotations**	**# of annotations**	**# of unique IDs**
**MIC training**	215	521	75
**MIC test**	254	375	53
**Bio-ID training**	63	156	13
**Bio-ID test**	9	20	6

## Results

### Evaluation metrics and characteristics of corpora

We used the micro-average precision (P), recall (R) and F-measure (F) to report the performance of the proposed method on two datasets. The first dataset is the extended MIC corpus described in the previous section. Since we mainly focused on the task of miRNA identification, we only evaluated the performance of our SPBA-based method on the annotations for the specific miRNAs.

The second evaluation corpus is the dataset released by the Bio-ID track. We used the corpus to study the challenges of recognizing and normalizing miRNAs mentioned in figure captions. The dataset was prepared as a part of the EMBO SourceData project (http://sourcedata.embo.org/), which contains documents in the BioC (32) format with figure captions collected from full-length articles along with annotations for multiple bio-entities. MiRNA was one of the entity types annotated in this dataset, and the annotations include their spans in figure captions and their corresponding Rfam IDs.

Table 2 summarizes the statistics of the annotated miRNAs in both corpora used in this study. MIC is the bigger corpus when compared to the Bio-ID.

### Performance on the MIC

Table 3 displays the entity recognition and normalization performance of the SPBA on the extended MIC corpus. For the recognition task, our method achieved satisfactory PRF scores on both the training and test sets and outperformed the performance reported by Bagewadi *et al*., which relied on the regular expressions for recognizing miRNAs. For the normalization task, our method also obtained satisfying F-scores.

**Table 3:** Entity recognition and normalization performance on the MIC

		**Training corpus**		**Test corpus**	
**Method**		**Bagewadi**	**SPBA**	**Bagewadi**	**SPBA**
**Recognition**	**P**	0.921	0.994	0.936	0.986
	**R**	0.928	0.990	0.934	0.991
	**F**	0.924	0.992	0.935	0.988
**Normalization**	**P**	n/a	0.994	n/a	0.986
	**R**	n/a	0.984	n/a	0.878
	**F**	n/a	0.989	n/a	0.928

### Performance on the Bio-ID corpus

Table 4 shows the performance of entity recognition and normalization on the training and test sets of the Bio-ID track. Using the official evaluation script provided by the Bio-ID organizers, we reported the identification performance in terms of micro-PRF scores under the strict matching mode that considers the boundary of a recognized miRNA exactly matching that of the reference annotation as a true positive.

**Table 4:** Performance on the Bio-ID dataset

	**Train set**			**Test set**		
Task	P	R	F	P	R	F
Recognition	0.325	1.00	0.491	0.085	1.00	0.145
Normalization	0.253	0.865	0.373	0.067	0.85	0.125

We can see that the developed method achieved recalls of 0.865 and 1.00 with very low precisions (0.253 and 0.067) resulting in frustrating F-scores of 0.373 and 0.125 on the Bio-ID training and test datasets, respectively.

### RESTful web service

A Representational State Transfer (RESTful) web service (16) for the developed miRNA identification component was implemented according to the protocol defined by the Biomedical annotation meta-server (BeCalm) platform (http://www.becalm.eu/) (33). Our RESTful service consists of three major components. The first is the data retrieval component that can retrieve articles from remote data sources. In our current implementation, four data sources are supported. Two of the data sources are PubMed Central and PubMed fetched by using the NCBI E-utilities. The other two are the pattern server and the abstract server released in the BioCreative V.5 technical interoperability and performance of annotation servers (TIPS) task (33).

The core of the RESTful service is our SPBA-based miRNA identification component. For a given article, MedPost (34) was used to split the text into sentences and generate tokens for each sentence. We then employed our SPBA-based miRNA identification method to recognize miRNA mentions in the pre-processed sentences and normalized them with corresponding Rfam IDs. The last component is the BeCalm communication module, which receives requests from the BeCalm platform, checks the correctness of the authentication key provided in each request, authorizes the requests and then responds to BeCalm with an acknowledgement message. All approved requests were sent to the first component for downloading articles from remote data sources. The downloaded articles are then processed by the core of our service for miRNA identification. Finally, the identified miRNAs are encoded in the JavaScript Object Notation (JSON) format defined by the TIPS task and sent back to the BeCalm platform.

The developed service has been employed on the text mining pipeline (35) developed for miRTarBase (36) to semi-automatically curate experimentally validated miRNA target genes from literatures.

## Discussion

### Ambiguous lexical entries

As described in the Methods section, we compiled a lexicon for normalizing the recognized miRNAs from the family file provided by the Rfam database. To assess the ambiguity of the lexicon, we transformed the names to generate variations by replacing ‘-’ and ‘_’ with whitespace characters and converted all letters to lower case. Furthermore, we explored a list of English words obtained from the Moby lexicon project website (the website of the Moby project is available at http://icon.shef.ac.uk/Moby/) to identify English words so that we could distinguish miRNA names that were ambiguous with general English terms.

On average, each miRNA name is associated with 1.022 IDs, while each ID is linked to 2.969 names. The ambiguity of miRNA names with general English terms is 0.3%, which falls into the range of the ambiguities of gene names in the four individual organisms ranging from 0 to 2.4% (37). MiRNA names as such like hammerhead and bantam does not cause difficulties in recognizing miRNAs that exist in both corpora examined in this study because there were no observed instances. Furthermore, unlike miRBase (38), the primary repository for published miRNA sequences and annotation data, the records in the Rfam database are not organism specific. Therefore, inter-species ambiguity is not an issue in this task. This is the reason that the performances of normalization are very close to that of recognition in both Tables 3 and 4.

**Figure 3:**
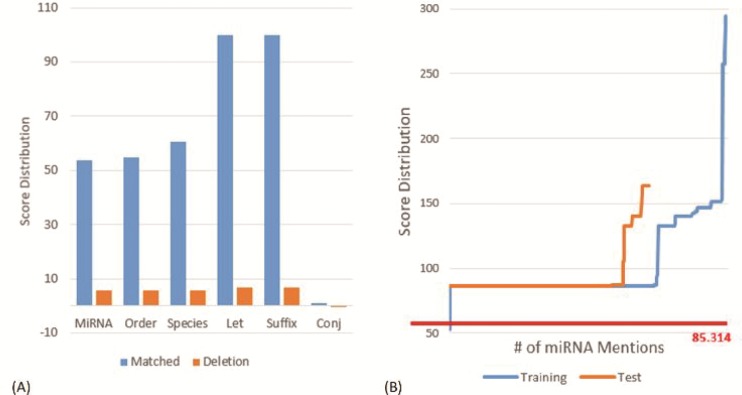
**(A)** Score distribution of the defined slots on the MIC training set. **(B)** Score distribution of the matched principles on the MIC corpus. The y-axis is the score for the considered miRNA mention. The x-axis is the number of the considered miRNA mentions. We sorted the scores of all recognized miRNAs in ascending order before plotting the chart.

**Table 5:** Inconsistent annotations observed in the Bio-ID corpus

**Type**	**Example**	**Frequency**
**Missing label**	Expression of miRNAs **miR-146a** and **miR-505** correlated with … The two most highly expressed miRs (**mir-21-5p** and **miR-146b-5p**) are also indicated.	168
**Non-miRNA**	TarA, 7SK, SgrS, GadY, HhR, U1, U2, U6, U42	104

### Analysis of the generated slots and principles

Figure 3A depicts the distribution of scores on the MIC training set for the defined slots in matched and deletion cases. As one can see that the slots ‘Let’ and ‘Suffix’ have the highest matching score of 100, which indicates that both slots only appeared within the miRNA mentions in the MIC training set. The ‘Let’ slot was defined for the precursor miRNAs that comprises key terms such as ‘let’ for the family of the lethal-7 gene and ‘lin’ for the lin-4 precursor. The ‘Suffix’ slot includes terms like ‘3p’ and ‘5p’. Furthermore, we noticed that the ‘Conj’ slot that contains conjunctions such as ‘and’ has a very low matching score of 0.972 and a negative deletion score (−0.041). The slot was generated by our algorithm because in the MIC training set there are miRNAs described by using conjunctions like ‘miR-107, -130a, -223, -292-5p, -433-3p, -451, -541, and -711’.

The commas shown in the miRNA mention above are examples of insertions. The insertion score for comma was estimated as −25.86 based on the MIC training set. In addition to the insertion of the defined slots, the insertion of ‘-’ has the least negative score of −1.030, while the top three negative insertion scores observed in the dataset were for the symbols ‘)’, ‘(’ and ‘,’.

Figure 3B shows the score distributions generated by the proposed SPBA method on the MIC corpus. The red line is the threshold of the dominant principle shown in Table 1. The highest score was observed on the MIC training set with a value of 294.6 for the mention ‘miR-21, 221, 128a, 128b, 128c, 181a, 181b, 181c’. The ability to recognize this long, variable length mention demonstrated the power of SPBA. In order to identify mentions as such, pattern-based approaches need to compile complex patterns to capture all of the possible variations written by authors. By contrast, SPBA simply relied on the dominant pattern demonstrated in Table 1 along with the matching score to determine whether or not to accept the matched instance. For this lengthy mention, the matched slots include ‘miRNA’ and two ‘Order’ slots along with seven insertions of ‘,’, six insertions of the ‘Order’ slot and one insertion of ‘-’. This matching process is also applied to mentions like ‘microRNA (miRNA)-146a’, ‘miR-29a/b-1’ and other variations.

On the other hand, the lowest score shown in Figure 3B is 53.8, which was actually filtered out by our SPBA. The annotated mention is ‘miR’ that only matched our ‘miRNA’ slot. The entity is described in the following sentence:

‘*… miR-17/92 is a positive effector of Shh-mediated proliferation and that aberrant expression/amplification of this **miR** confers a growth advantage …*’.

In this sentence, miR is an anaphora referring to miR-17/92. However, recognizing these terms is meaningless without implementing co-reference resolution.

### Error analysis

As illustrated in Table 4, our method had an optimal recall for recognition on both the training and test sets, but the precisions were rather low. After analyzing the dataset of the Bio-ID track, we observed that the majority of the errors were due to inconsistent annotations. For instance, the mention U2 (Rfam:RF00004) existed several times in the figure 4 of the article (PMC4801943) but was not annotated in the corpus. However, our method recognized and normalized this entity after we retrained our SPBA method on the Bio-ID training corpus. Some false negatives were caused by the usage of abbreviated terms in the figure captions. For example, the term ‘HhR’ mentioned in the figure captions of the article PMID 27009120 refers to a self-cleaving hammerhead ribozyme that generated an mRNA reporter with a 30-end in Drosophila melanogaster cells. The full name (hammerhead ribozyme) of the abbreviated term is only available in the Results section of the article. Unfortunately, the current implementation does not consider the information from the full text.

Nevertheless, if we take a closer look at these cases, we can notice that U2 is a small nuclear RNA that is recruited in the splicing biological process, and HhR is a distinct RNA motif that catalyzes specific biochemical reactions, so they are indeed not miRNAs. We summarized the types of the inconsistent annotations observed on the Bio-ID corpus in Table 5. Note that the analysis was based on the output of our SPBA method. The observation indicates that the Bio-ID corpus is not a reliable corpus for evaluating the performance of miRNA identification at the current stage.

## Conclusion

In this study, we have developed a method based on the statistical principle for miRNA identification. The proposed method combines the advantages of supervised learning and pattern-based approaches to provide an integrated solution for recognizing miRNAs mentioned in free text and normalized them to the corresponding IDs in the Rfam database. The two major advantages of the proposed method are (i) the knowledge learned from the corpus is organized in a human-interpretable manner to understand the reason why the system makes such decision and can also be further enhanced by domain experts, and (ii) the proposed scoring mechanism along with the idea of IDS enables our method to use a dominant principle to recognize a variety of miRNA mentions. Furthermore, we compiled a corpus for normalizing miRNAs to the Rfam database and analyzed the ambiguity level for normalizing miRNA to Rfam. We observed that on average each miRNA name is associated with 1.022 IDs while each ID is linked to 2.969 names, and the degree of ambiguity with general English terms is similar to that of gene names. Fortunately, the data collected in the Rfam database is not organism specific. Therefore, we did not encounter the inter-species ambiguity issue in this study. We believe that normalizing miRNAs to the primary repository for published miRNA sequence, such as miRBase, should be more challenging.

MiRNAs play a vital role as prognosis biomarkers in the early detection of various diseases. Scientific literature related to methods of identifying, isolating and amplifying miRNAs and potential use of miRNAs as biomarkers for multiple cancer types are therefore increasing rapidly. To facilitate better understanding and keep up to date on the latest advancements and applications of miRNAs, a systematic automated methodology that can identify miRNAs mentioned in unstructured text is essential. The results and findings presented in this study provide useful insights into the challenges associated with miRNA recognition and normalization using IE methods that need to be further investigated in future studies.

## Supplementary Material

Supplementary_Material_baz030

## Notes

**Database URL**: https://bigodatamining.github.io/software/201901/
