# Supplementary material for: Statistical principle-based approach for recognizing and normalizing microRNAs described in scientific literature
Source: Database (Oxford). 2019 Feb 27;2019:baz030. doi: 10.1093/database/baz030 (PMC6391575; doi:10.1093/database/baz030)
Supplement: Supplementary_Material_baz030 [file supplementary_material_baz030.docx]

# miRNA Normalization Corpus

We extended the miRNA interaction corpus annotated by Bagewadi, Bobic, Hofmann-Apitius, Fluck, and Klinger (2014) by including normalization annotations for miRNAs. The corpus contains 301 abstracts divided into training and test sets. There are 1,864 sentences in the training set and 780 sentences in the testing set. Five bio-entity types, including specific-miRNA (e.g. has-miR-124b), non-specific-miRNA (e.g. microRNAs or miRNAs), disease, gene, and species, were annotated. All of these annotations were annotated at the sentence level. The detail statistics of the corpus is shown in Table I. In the training set, 327 sentences contain 529 specific miRNAs. And 376 specific miRNAs are included in the testing set.

1. annotated entities statistics of the miRNA Interaction corpus.

| Annotation Class | Corpus | |
| --- | --- | --- |
|  | Training | Test |
| **Non-specific MiRNAs** | 1,170 | 336 |
| **Specific MiRNAs** | 529 | 376 |
| **Genes/Proteins** | 734 | 324 |
| **Diseases** | 1,522 | 640 |
| **Species** | 546 | 182 |
| **Sentences** | 1,864 | 780 |
| **miRNA Entities** | 1,699 | 712 |

Table II shows the statistics of our extended annotations. The training data set consisted of a total of 521 annotated miRNAs corresponding to 75 unique IDs from 1,863 sentences from 201 articles. The test data set in total consisted of 780 annotated sentences from 100 articles with 375 annotations and 53 unique IDs. Some miRNA annotations were annotated with more than one ID, such as “RF00103, RF00446” for the mention “miR-1/133a”.

1. Statistics of the Annotated Rfam IDs in the miRNA Interaction corpus.

| Dataset | # of sentences with miRNAs annotations | Total # of annotations | # of unique IDs |
| --- | --- | --- | --- |
| **Training** | 215 | 521 | 75 |
| **Test** | 254 | 375 | 53 |

The annotated corpus is available at the project web site on <https://bigodatamining.github.io/software/201901/>. The direct download link is <https://bigodatamining.github.io/software/corpus/mic_rfam_v1.0.zip>

# Inconsistent Annotations in the Bio-ID Corpus

Table III summarizes the inconsistent annotations observed in the training set of the Bio-ID corpus. Here we followed the two types defined in our paper to categorize the inconsistency. Note that we only listed one instance of the inconsistent annotations even if that instance appeared several times in the document.

1. Inconsistent Annotations Observed in the Bio-ID Training Set

| Digital Object Identifier | Inconsistent Type | Case | Recommend ID |
| --- | --- | --- | --- |
| 10.1038/ncomms2090 | Missing Label | miR-132 | RF00662 |
| 10.1038/embor.2013.53 | Missing Label | lsy-6 | RF00823 |
| 10.1038/ncb2611 | Missing Label | miR-16 | RF00254 |
| 10.1038/ncb2611 | Missing Label | let-7 | RF00027 |
| 10.15252/embj.201490441 | Missing Label | miR-17 | RF00051 |
| 10.15252/emmm.201404511 | Missing Label | miR-210 | RF00679 |
| 10.15252/emmm.201505495 | Missing Label | miR-10 | RF00104 |
| 10.15252/embj.201593113 | Non-miRNA | U1 |  |
| 10.15252/embj.201593113 | Non-miRNA | U2 |  |
| 10.15252/embj.201593113 | Non-miRNA | U6 |  |
| 10.15252/embj.201593113 | Non-miRNA | U42 |  |
| 10.15252/embr.201540970 | Missing Label | miR-515 | RF00639 |
| 10.15252/embj.201592901 | Non-miRNA | HhR |  |
| 10.15252/embj.201592901 | Missing Label | miR-1 | RF00103 |
| 10.15252/emmm.201506031 | Missing Label | miR-29 | RF00074 |
| 10.15252/emmm.201506031 | Missing Label | miR-30 | RF00131 |
| 10.15252/emmm.201505925 | Missing Label | miR-150 | RF00767 |
| 10.15252/embr.201541930 | Missing Label | miR-21 | RF00658 |
| 10.15252/embr.201541930 | Missing Label | miR-24 | RF00178 |
| 10.15252/embr.201541930 | Missing Label | miR-122 | RF00684 |
| 10.15252/embr.201541930 | Missing Label | let-7 | RF00027 |
| 10.15252/embj.201694857 | Missing Label | let-7 | RF00027 |
| 10.15252/embj.201694056 | Missing Label | miR-34 | RF00456 |
| 10.15252/embj.201694056 | Missing Label | miR-449 | RF00711 |
| 10.15252/embr.201540678 | Missing Label | miR-10 | RF00104 |

# References

Bagewadi, S., Bobic, T., Hofmann-Apitius, M., Fluck, J., & Klinger, R. (2014). Detecting miRNA Mentions and Relations in Biomedical Literature. *F1000Res, 3*, 205. doi:10.12688/f1000research.4591.3
